# A Case of Campylobacter Fetus Subspecies Fetus Systemic Infection

**DOI:** 10.7759/cureus.23963

**Published:** 2022-04-08

**Authors:** Pabitra Adhikari, Drashti Antala, Birat Bhandari, Khalid Mohamed, Goar Egoryan, Jonathan J Stake, Harvey Friedman

**Affiliations:** 1 Internal Medicine, AMITA Health Saint Francis Hospital, Evanston, USA; 2 Infectious Disease, AMITA Health Saint Francis Hospital, Evanston, USA; 3 Critical Care, AMITA Health Saint Francis Hospital, Evanston, USA

**Keywords:** bacteremia, synovial fluid, septic arthritis, immunocompromised hosts, campylobacter fetus subspecies fetus (c fetus)

## Abstract

*Campylobacter* are gram-negative bacilli commonly known to cause gastro-intestinal infection; however, species like *Campylobacter fetus subspecies fetus (C. fetus)* have been documented to cause severe systemic illness, especially in immunocompromised hosts. It has been linked with severe sepsis, septic arthritis, endocarditis, and subdural abscess. We report a case of a 65-year-old male with a history of human immunodeficiency virus infection (HIV) and chronic hepatitis B presenting with high fevers, pain, and swelling in multiple joints. His blood cultures grew *C. fetus*. Synovial fluid analysis from the knee joint revealed leukocytes of 17,000 with 93% neutrophils, and gram stains and cultures from synovial fluid were negative. The patient improved with piperacillin-tazobactam and vancomycin which were transitioned to amoxicillin-clavulanic acid and azithromycin as the patient returned to baseline functional status.

## Introduction

*Campylobacter *bacilli are known for causing diarrheal illness secondary to consumption of contaminated poultry or unpasteurized dairy products. In rare instances, especially in immunocompromised patients, some species can cause bacteremia and involve systemic organs. It can affect previously damaged joints or prosthetic joints and lead to septic arthritis. Septic arthritis is usually monoarthritis and in a few instances can involve multiple joints especially when the joints are affected by previous or concomitant inflammatory arthritis. We report a case of a 65-year-old patient with a history of alcohol abuse, HIV, and chronic untreated hepatitis B who presented with high fever and pain and swelling in multiple joints.

## Case presentation

A 65-year-male with a past medical history of alcohol and substance abuse, HIV on highly active antiretroviral therapy (HAART), and chronic hepatitis B presented to the emergency department with one day of high fevers and multiple joint pains. He denied any recent gastrointestinal disturbances. Vital signs were significant for a fever of 103F. On physical examination, the joints were warm, erythematous, and swollen with severe tenderness on the left knee and the right ankle with restricted range of motion of these joints. The patient was started empirically on piperacillin-tazobactam and vancomycin. 

Laboratory work (Table 1) revealed leukocyte counts of 15000/mL with neutrophils 84.9%, C reactive protein (CRP) of 22.7 mg/dl, procalcitonin of 0.70 ng/dl. CD4 count percentage was 11% with an absolute count of 159/cu mm, and HIV viral load was 149 copies/ml. X-ray of the left knee showed a small effusion in the knee joint. Blood culture grew *C. fetus *on three subsequent vials. The synovial fluid analysis (Table 2) revealed a WBC count of 17,000/cu mm and a neutrophil count of 93%. However, no growth of bacteria was observed in synovial fluid gram stain and culture nor was any crystals seen. The diagnosis of possible polyarticular septic arthritis was considered given the clinical features of high fever, swollen and tender joints with neutrophil predominant synovial fluid. The patient experienced symptomatic joint pain relief and could bear weight following three days of empiric antibiotics along with the improvement of leukocytosis. Vancomycin and piperacillin-tazobactam were transitioned to oral amoxicillin-clavulanic acid based on the culture sensitivity report and were continued for a total duration of six weeks. Transthoracic echocardiogram was negative for valvular vegetations. His Hepatitis B (HBV) viral load was more than 10,000,000 copies. Other workups for infection including GC chlamydia probe and HCV were negative.

**Table 1:** Significant laboratory findings (blood) suggestive of infection mL: Milliliters, dL: Deciliters

Significant laboratory workup (Blood)	Results	Reference range
White blood cells (WBCs)	15,000 cells/mL	4,000 to 10,000 cells/mL
Polymorphonuclear neutrophils (PMNs)	84.9%	40 to 60 %
C Reactive Protein (CRP)	22.7 mg*/*dL	Less than 10 mg/dL
HIV Viral load	149 copies/mL	Less than 40 copies/mL

**Table 2:** Synovial fluid analysis findings mL: Milliliters

Synovial fluid analysis findings	Results	Reference range
WBCs	17,000 cells/mL	Less than 200 cells/mL
Polymorphonuclear neutrophils (PMNs)	93%	Less than 25%

## Discussion

*Campylobacter* genus is a group of small gram-negative curved motile bacteria known to cause food-borne illness [1]. Most cases present as diarrhea and only about 0.15% of them can lead to bacteremia and involve distant organs. About 90% of the *campylobacter* diarrheal illness is caused by* C. jejuni* or *C. coli*, whereas a small number of cases are due to *C. fetus *[2]. On rare occasions, it can cause invasive, potentially fatal infections in immunocompromised individuals [1,3]. *C. fetus* is the species most frequently associated with systemic campylobacteriosis and is usually isolated in immunocompromised individuals [3]. In 24 to 41% of cases, it was found to cause septicemia without apparent localized infection [1]. Immunosuppression (from HIV, hematological malignancy, splenectomy), abnormal heart valves, diabetes, prosthesis, and liver disease can predispose to* C. fetus* bacteremia [2].

*C. fetus* possesses a surface layer protein (SLP) that functions as a capsule and protects from the bactericidal action of normal serum and also undergoes antigenic variation helping it to persist in human or animal hosts and accounts for recurrent infection despite the appropriate antibiotic therapy [3]. Due to the SLP, it is resistant to opsonization and can lead to systemic infection, and involves multiple organs causing osteomyelitis, lung abscess, arthritis, neurological infections, subdural abscess, and cholecystitis [1,4]. Septic arthritis linked to prostheses has also been also described [4,5]. Cattle and sheep-derived food products are most likely transmission routes [2]. 

Septic arthritis generally presents as monoarthritis with acutely swollen, red, and tender joints [6]. In rare instances, when the joints have been affected by prior polyarticular inflammatory arthritis, it can present as polyarthritis [6]. Such a presentation can delay the diagnosis and make the distinction more difficult [6]. About 15% of septic arthritis cases have been reported to be polyarticular with the number of mean affected joints being three [7]. The knee joint was the most common joint involved followed by the elbow, shoulder, and hip [7].

Synovial fluid analysis is the first step in diagnosing joint infection. Synovial fluid analysis with a white blood cell count greater than 50,000 and 90% neutrophil predominance is consistent with a diagnosis of septic arthritis. However, some cases may have a WBC count of less than 50,000/mL, especially in immunocompromised patients with reduced leukocyte response [6]. In a retrospective study, Baran et al. found that the percentage of neutrophils in white blood cells (WBCs) differentials was a more sensitive predictor for non-prosthetic septic arthritis compared to the absolute WBC count in the synovial fluid analysis [5, 8]. Culture and gram stain will confirm the infection but a negative result can occur if a patient receives antibiotics prior to arthrocentesis or if the fluid sample is inadequate [6].

*C. fetus* has been reported to be linked with septic arthritis. Systemic illness from* C. fetus *may be preceded by diarrheal illness in some instances. Yoa et al. and Zamora-López et have described the cases of prosthetic joint infection in immunocompromised patients who did not have any gastrointestinal symptoms like our patient [4,5]. In a case series by Meyer et al.,* C. fetus *was found to be responsible for osteoarticular or cutaneous infection in patients with rheumatoid arthritis treated with rituximab [9]. Another campylobacter species, *C. coli* has been linked to causing prosthetic joint infection following consumption of uncooked chicken wings [10].

Our patient was immunocompromised with HIV infection and presented with acute pain in the left knee joint. He had concomitant acute pain in his right ankle and bilateral elbow joints. He had associated fever and myalgia. Based on the clinical evaluation, the differential diagnosis included septic arthritis (monoarticular or polyarticular), viral arthritis, gout, pseudogout, or post-infectious reactive arthritis. It has been found that when a patient receives antibiotics before the arthrocentesis, leukocytosis of greater than 16,000 should be treated as septic arthritis although WBC greater than 33,000 in patients who have not received antibiotics yields greater accuracy of diagnosis [10]. It is possible that due to the administration of antibiotics before synovial fluid analysis, the WBC was lower than 50,000 but the neutrophil predominance and systemic features with persistent *C. fetus* bacteremia were consistent with *C. fetus *as the culprit for septic arthritis in our patient.

## Conclusions

*C. fetus* is an uncommon cause of joint infection in immunocompromised patients. Systemic infection may be preceded by nonspecific gastrointestinal illness in some but not all cases. Appropriate history should be obtained for animal exposure and food intake. Timely arthrocentesis before antibiotic initiation is necessary and should not be delayed as it can confirm the diagnosis. Persistent bacteremia with* C. fetus* should prompt to rule out cardiac involvement as it has been reported to cause endocarditis.
